# Direct Detection and Sequencing of Damaged DNA Bases

**DOI:** 10.1186/2041-9414-2-10

**Published:** 2011-12-20

**Authors:** Tyson A Clark, Kristi E Spittle, Stephen W Turner, Jonas Korlach

**Affiliations:** 1 Pacific Biosciences, 1380 Willow Rd, Menlo Park, California, USA

**Keywords:** DNA Damage, Modified Bases, Sequencing

## Abstract

Products of various forms of DNA damage have been implicated in a variety of important biological processes, such as aging, neurodegenerative diseases, and cancer. Therefore, there exists great interest to develop methods for interrogating damaged DNA in the context of sequencing. Here, we demonstrate that single-molecule, real-time (SMRT^®^) DNA sequencing can directly detect damaged DNA bases in the DNA template - as a by-product of the sequencing method - through an analysis of the DNA polymerase kinetics that are altered by the presence of a modified base. We demonstrate the sequencing of several DNA templates containing products of DNA damage, including 8-oxoguanine, 8-oxoadenine, O6-methylguanine, 1-methyladenine, O4-methylthymine, 5-hydroxycytosine, 5-hydroxyuracil, 5-hydroxymethyluracil, or thymine dimers, and show that these base modifications can be readily detected with single-modification resolution and DNA strand specificity. We characterize the distinct kinetic signatures generated by these DNA base modifications.

## Background

DNA is under constant stress from both endogenous and exogenous sources. As the carrier of genetic information, DNA relies on the maintenance and repair of existing molecules and is the only biological molecule to do so. The bases exhibit limited chemical stability and are vulnerable to chemical modifications through different types of damage, including oxidation, alkylation, radiation damage, and hydrolysis. DNA base modifications resulting from these types of DNA damage are wide-spread and play important roles in affecting physiological states and disease phenotypes (reviewed in [1-3]). Examples include 8-oxoguanine, 8-oxoadenine (oxidative damage; aging, Alzheimer's, Parkinson's), 1-methyladenine, 6-O-methylguanine (alkylation; gliomas and colorectal carcinomas), benzo[a]pyrene diol epoxide (BPDE), pyrimidine dimers (adduct formation; smoking, industrial chemical exposure, UV light exposure; lung and skin cancer), and 5-hydroxycytosine, 5-hydroxyuracil, 5-hydroxymethyluracil, and thymine glycol (ionizing radiation damage; chronic inflammatory diseases, prostate, breast and colorectal cancer).

Currently, methods for detecting these and other products of DNA damage are limited to bulk measurements including chromatographic techniques, polymerase chain reaction assays, the Comet assay, mass spectrometry, electrochemistry, radioactive labeling and immunochemical methods (reviewed in [4]). To our knowledge, the integration of DNA damage detection into a high-throughput DNA sequencing technique has not been reported. Because base damage can occur at random DNA template positions, sequencing capabilities reaching the level of individual DNA molecules are highly desirable.

Recently, single-molecule, real-time (SMRT) DNA sequencing has been described for the direct detection of methylated and hydroxymethylated DNA bases [5]. In SMRT sequencing, the progression of single molecules of DNA polymerase is monitored in real time during base incorporations using fluorescent phospholinked nucleotides [6,7]. The dynamics of DNA polymerization is thereby recorded in the form of a train of fluorescent pulses. The length of time that the polymerase retains a nucleotide bound in its active site (pulse width, PW), and the time interval between successive nucleotide-bound states (interpulse duration, IPD) are the principal pulse metrics used in the analysis to ascertain that the polymerase kinetics was altered in the modification-containing template when compared to an unmodified control template [5]. Because the DNA polymerase is in contact with the modified base over a region of ~11 bases [8], the kinetic effects are not necessarily restricted to the nucleotide incorporation opposite of the modified base. The magnitude and extent of the kinetic signature is dependent on the type of the base modification and the local sequence context. This results in distinct, more complex kinetic signatures that can be used to discriminate between different chemical base modifications [5].

Here, we apply SMRT DNA sequencing towards the direct detection of damaged DNA bases. Using synthetic DNA templates, we survey several common products of DNA damage for their kinetic effects on the polymerase kinetics in SMRT sequencing. We show that this method can readily detect damaged DNA bases with single-base resolution and discriminate between different types of DNA damage products through distinct kinetic signatures observed for different damaged bases.

## Results and Discussion

To investigate the effect of products of DNA damage in SMRT sequencing, we designed synthetic SMRTbell™ templates [9] carrying two instances of a particular chemical base modification that can occur as a result of DNA damage (Additional File 1). The DNA templates were subjected to standard SMRT DNA sequencing [7] and analyzed for their effects on the polymerase kinetics, as compared to a control DNA template of identical sequence but lacking the two instances of chemical base modifications and containing the unmodified canonical bases at those positions. In the simplest form of analysis, the ratio of averaged kinetic values between the modified and the control template is calculated for each template position. A deviation of IPD or PW from a ratio of unity indicates the presence of a modified nucleotide [5]. For the characterization of products of DNA damage in the synthetic templates studied here, we employed this ratio analysis.

### Oxidative Damage

**Figure 1:**
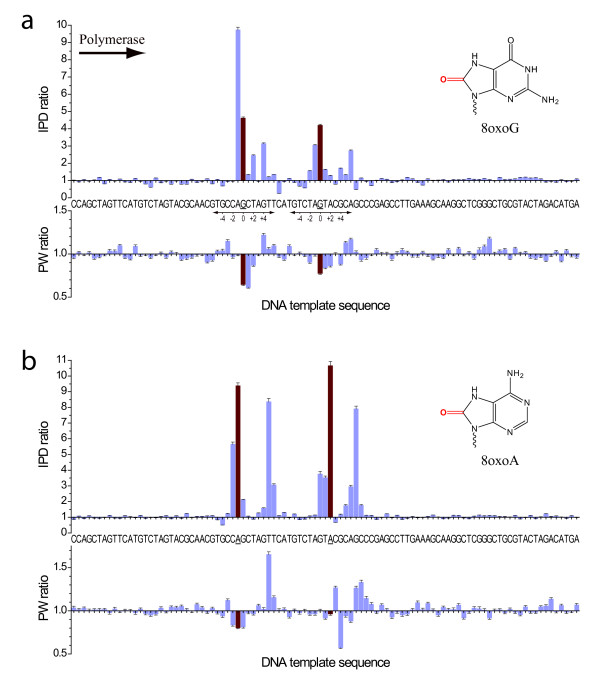
**Kinetic effects of products of oxidative DNA damage in SMRT sequencing**. Normalized IPD ratios (top panel) and PW ratios (bottom panel) between the modified and unmodified DNA templates are shown for (a) 8oxoG and (b) 8oxoA, with the DNA template sequence as the x axis. The two template positions carrying the base modifications are highlighted with red bars and underlined sequence labels. The direction of DNA polymerization (left to right, thus the DNA template sequence is 3' to 5') as well as the coordinate space relative to the modified sites as referred to in the text is indicated in (a) and omitted for clarity in the rest of the figures.

Products of oxidative DNA damage are very common, especially in mitochondrial DNA, and can accumulate with age, or in conjunction with diseases such as Alzheimer's, Parkinson's, and cancer [10-12]. We investigated the effects of 8-oxoguanine (8oxoG) and 8-oxoadenine (8oxoA) in SMRT DNA sequencing, and both resulted in readily detectable kinetic signatures (Figure 1). The presence of 8oxoG caused a ~4fold increase of the IPD at the sites of the base modification (position zero), compared to the unmodified control (Figure 1a). This corresponds to a ~4fold delay of the binding of the phospholinked dCTP nucleotide into the active site across the 8oxoG modification in the template. Additional characteristic kinetic signals surrounding the site of the base modification were observed, with a relatively strong IPD ratio increase for nucleotide incorporations just prior to the site of the modification (position -1), and smaller signals up to 6 bases past the modification site (positions +1 to +6). For both modification sites, a decrease in the IPD ratio for the +7 position was observed, i.e. the polymerase bound the nucleotide faster compared to the unmodified control. Pulse widths were also affected by the 8oxoG modifications albeit with smaller magnitude, with decreased PW values spanning positions zero to +2 compared to the control. In addition to the kinetic signature, we observed an increased frequency of misincorporation of phospholinked dATP opposite the 8oxoG template position, ~5fold higher compared to the control template (data not shown). This is not unexpected since 8oxoG is known to mispair with adenine [13].

The kinetic signature of 8oxoA (Figure 1b) was overall similar to 8oxoG, but with a stronger, ~10fold increased IPD at the modification position, and additional signals one to two bases prior to the modification position. In the region five to seven nucleotide incorporations after the 8oxoA position, strong additional kinetic signals were observed, exhibiting similar magnitudes compared to the modification position IPD ratio. Pulse widths were slightly increased in this region. The observed kinetic signature for 8oxoA differs from the previously described kinetic signature for adenine methylation [5]; N6-methyladenine (6mA) exhibited kinetic signals at the zero position of ~5fold, i.e. half the signal observed for 8oxoA, and a weak signal at the +5 position of ~2fold. These differences can be used to discriminate between different chemical base modifications that occur on the same base type.

### Alkylation

**Figure 2:**
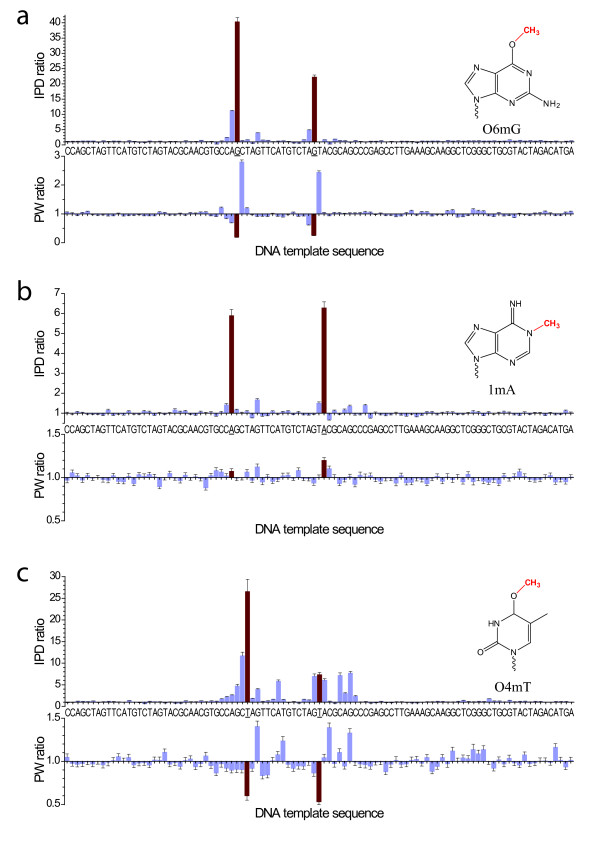
**Kinetic effects of products of alkylation DNA damage in SMRT sequencing**. Normalized IPD ratios (top panel) and PW ratios (bottom panel) between the modified and unmodified DNA templates are shown for (a) O6 mG, (b) 1 mA, and (c) O4 mT, with the DNA template sequence as the x axis. The two template positions carrying the base modifications are highlighted with red bars and underlined sequence labels.

The transfer of an alkyl group onto a DNA base can occur naturally for epigenetic modifications, but also detrimentally as part of DNA damage, and it is also used in chemotherapy to damage the DNA of cancer cells (reviewed in [2]). We studied the kinetic effects of three examples of alkylated bases in SMRT sequencing: O6-methylguanine (O6mG), 1-methyladenine (1mA), and O4-methylthymine (O4mT) (Figure 2). The presence of O6mG resulted in a very strong kinetic signal at the modification position, with IPD ratios of ~20-40, accompanied by a weaker signal at the -1 position (Figure 2a). Pulse widths were also markedly affected, with decreased PW ratios at the -1 position and more strongly at position zero, and an increased PW of ~2-3fold at the +1 position. DNA templates containing 1mA displayed IPD ratios of ~5-6 at position zero, a smaller signal at the -1 position, and no effects on the PW (Figure 2b). The kinetic effects of 1mA are therefore similar to those of 6mA [5]. O4mT modifications (Figure 2c) resulted in ~10fold or greater IPD ratios at the modification position, additional signals two bases up to two bases prior and after the modification position, and at positions +4 to +6. Pulse widths were reduced at position zero, and elevated at positions +2, +6 and +7.

### Ionizing radiation

**Figure 3:**
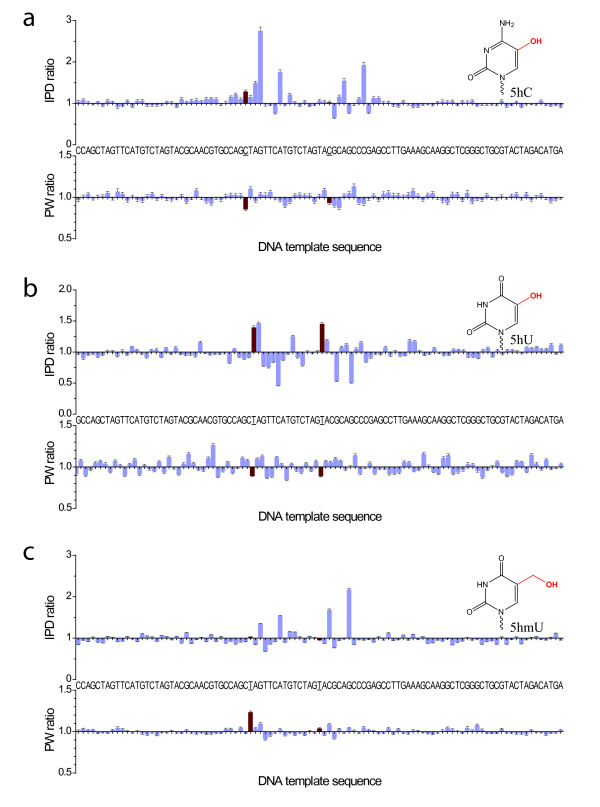
**Kinetic effects of products of ionizing radiation DNA damage in SMRT sequencing**. Normalized IPD ratios (top panel) and PW ratios (bottom panel) between the modified and unmodified DNA templates are shown for (a) 5 hC, (b) 5 hU, and (c) 5 hmU, with the DNA template sequence as the x axis. The two template positions carrying the base modifications are highlighted with red bars and underlined sequence labels.

Ionizing radiation can confer damage to DNA bases either through direct effects, or indirectly through the generation of free radicals that elicit damage [14]. Products of DNA damage resulting from ionizing radiation include 5-hydroxycytosine (5hC), 5-hydroxyuracil (5hU), 5-hydroxymethyluracil (5hmU). Kinetic effects from these base modifications (Figure 3) were also clearly detectable albeit with smaller magnitudes, presumably due to the smaller size of these modifications, their altered position relative to affecting base pairing during nucleotide incorporation, smaller geometric distortions of the nascent double-stranded DNA molecule, and less contact of the major groove by the polymerase enzyme [8]. For 5hmU, the magnitudes and phenomenology of the kinetic effects were similar to what has previously been observed for the structurally related 5-hydroxymethylcytosine modification [5,15], with characteristic IPD signals at positions +2 and +6. Interestingly, for 5hC there are also two principal delayed locations, but in this case at positions +3 and +7. Thymine glycol, another principal DNA lesion induced by ionizing radiation and also oxidation, resulted in read termination, as the polymerase was not able to advance beyond the site of the modification (Additional File 2).

### UV radiation

**Figure 4:**
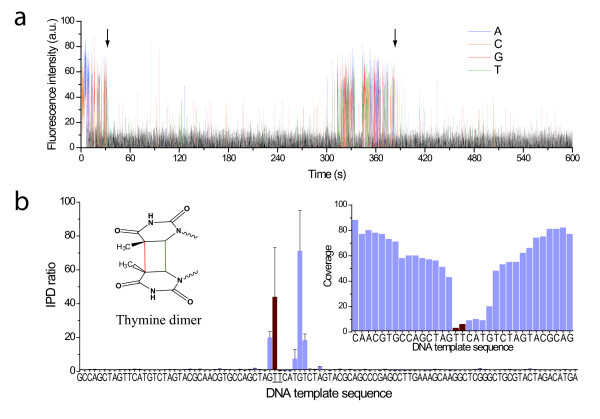
**Kinetic effects of products of UV radiation DNA damage in SMRT sequencing**. (a) Example sequencing trace, showing very long pausing by the polymerase upon repeated encounters with a thymine dimer (arrows) on the SMRTbell DNA template. (b) Normalized IPD ratios between the thymine dimer containing and unmodified control DNA templates. The template sequence is given as the x axis. The two template positions constituting the thymine dimer are highlighted with red bars and underlined sequence labels. The inset shows the lack of sequencing coverage after the thymine dimer position because of the extremely long pauses by the polymerase induced by the lesion.

Cyclobutane pyrimidine dimers are the most common form of DNA damage following UV radiation [1]. We investigated the effect of a thymine dimer in the DNA template during SMRT sequencing. We observed that the polymerase was able to bypass this lesion; however, the thymine dimer had dramatic effects on the polymerase kinetics with extremely long pauses around the site of the lesion, in many instances lasting several minutes (Figure 4a). Such severe pausing translated to very large IPD ratios (Figure 4b). It also affected the ability to accurately map the sequencing reads in the vicinity of the thymine dimer, resulting in reduced sequencing fold coverage around the position of the modification (Figure 4b, inset), and translating to a higher uncertainty in measuring IPD ratios near the lesion. The sequencing kinetics were very strongly affected at many positions along the region of contact between the thymine dimer and the polymerase (positions -1 to +8), likely due to geometric distortions imparted by the lesion. Upon passing the lesion, sequencing resumed normally until the next encounter on the same SMRTbell template molecule (Figure 4a).

## Conclusions

Replicative DNA polymerases have evolved to move along DNA templates, synthesizing a complementary DNA strand, with remarkable efficiency, speed and fidelity [16]. The presence of various forms of DNA damage in the template strand can lead to transient stalling, misincorporation, or even termination of DNA polymerization, depending on the type of damage [3,10,17]. Taking advantage of this exquisite sensitivity of polymerases towards changes in the DNA template, we have investigated the ability of SMRT DNA sequencing to detect products of DNA damage through an analysis of the polymerase kinetics that is recorded in real time by this method. We have demonstrated the detection of several common forms of DNA damage while performing the regular sequencing protocol, and without the need for special upfront sample preparation steps.

Damaged DNA bases can impart effects on the polymerase kinetics in several ways. IPDs can be affected by (i) changes in the affinity of binding the incoming nucleotide, or (ii) altered DNA translocation rates following the phospholinked nucleotide incorporation. Variations in PW can be caused from (i) effects on the rates of conformational changes of the enzyme, as well as (ii) the rate of catalysis during the nucleotide incorporation cycle, as the damaged base can distort active site geometries. All of these effects are captured in SMRT sequencing through the real-time monitoring of each nucleotide incorporation event, thereby making the method sensitive to even extremely small changes to relatively subtle chemical modifications, such as 5hC, 5hU or 5hmU. Because of the nature of the SMRTbell DNA template allowing the sequencing of both the forward and the reverse strand of the same DNA molecule during a SMRT sequencing reaction, products of DNA damage can be detected in a strand-specific manner, allowing for the differentiation of hemi- and fully-modified positions [15]).

Kinetic signals were not limited to just the modified base position, but were observed over a confined region surrounding the base modifications, encompassing approximately three nucleotide incorporations prior and seven incorporations after the site of the modification, consistent with the 'footprint' of the polymerase on DNA [8], and with previously described results for base methylation [5]. We attribute this effect to the extended contact the polymerase makes with the incoming DNA template and the nascent double-stranded DNA over its DNA binding region (Additional File 3). For misincorporation events, the transmission of the presence of the mismatch back to the enzyme active site through long-range distortions in the DNA has been described [18]. Similarly here, distortions in the DNA geometry by a modified base throughout the extended DNA binding region affect the dynamics of nucleotide incorporations at the polymerase active site, giving rise to additional signals that result in a distinct kinetic signature for a given damaged DNA base. This allows the discrimination of base modifications of different products of DNA damage when they occur on the same base type - potentially even on the same DNA molecule. For example, the kinetic signature of 8oxoA was readily distinguishable from methylated A, and likewise 8oxoG from O6mG, and O4mT from 5hU and 5hmU. We are in the process of incorporating these characteristics into bioinformatics algorithms for the automated calling of damaged DNA bases during SMRT sequencing, as well as extending these studies to demonstrate sequencing of modifications that are in very close proximity to each other on the same DNA strand.

Besides the sequencing application, the method opens potential paths to increase our understanding about how different types of DNA polymerases are affected by certain types of DNA lesions. The SMRT sequencing assay could potentially be adapted to study the detailed dynamics of lesion-specific polymerases at the single-molecule level, and to combine their activities with replicative polymerases to capture products of DNA damage which are currently resulting in read termination. We anticipate that SMRT sequencing will become a powerful, high-throughput tool for the detection and sequencing of DNA containing damaged bases to improve our understanding of aging, DNA damage-related diseases, DNA polymerase enzymology, DNA repair mechanisms, and chemotherapeutic efficacies. The presented method should also be applicable towards detecting previously unknown base changes in DNA.

## Methods

Custom oligonucleotides containing modified bases were purchased from Bio-synthesis (Lewisville, TX), Trilink BioTechnologies (San Diego, CA), ChemGenes (Wilmington, MA) and Integrated DNA Technologies (Coralville, IA). A list of the sequences can be found in Additional File 4. All oligonucleotides contained 5' phosphate groups. SMRTbell templates were generated by ligating several synthetic oligonucleotides, one of which contained two instances of a chemical base modification (Additional File 1). Complementary and hairpin oligonucleotides were annealed by heating to 80°C for 2 minutes and slowly cooling to 25°C (0.1°C/sec) in 10 mM Tris (pH 7.5), 100 mM NaCl. Annealed oligonucleotides were ligated using T4 DNA Ligase (NEB; Ipswich, MA) for 60 minutes at 25°C followed by heat kill for 10 minutes at 65°C. Incompletely formed SMRTbell templates were degraded with a combination of Exonuclease III (NEB; Ipswich, MA) and Exonuclease VII (USB; Cleveland, OH) at 37°C for 30 minutes. SMRTbell templates were purified using QIAquick PCR Purification columns (Qiagen; Valencia, CA).

SMRTbell templates were subjected to standard SMRT sequencing using an engineered phi29 DNA polymerase, as described [6,7]. All templates were run in duplicate on different days, different sequencing instruments, and using different reagent lots to verify the reproducibility of the reported results. Reads were processed and mapped to the respective reference sequences using the BLASR mapper (http://www.pacbiodevnet.com/SMRT-Analysis/Algorithms/BLASR) and the Pacific Biosciences SMRT Analysis pipeline (http://www.pacbiodevnet.com/SMRT-Analysis/Software/SMRT-Pipe) using the standard mapping protocol. IPDs were measured as previously described [5] for all pulses aligned to each position in the reference sequence. Baseline correction was applied by dividing the IPD mean for each position by the average of mean IPDs over all positions in the template, excluding the positions of base modifications and a window of six bases in each direction around such positions. In addition, 5% of outlier values were trimmed from both sides of the IPD distribution at each position before computing the mean. Thereafter, the ratio of mean IPDs was computed between the modified and control template samples for each template position.

The uncertainty in the IPD ratio was quantified by calculating the standard error of the mean of the IPD ratio using the delta method:

$$\hat{SE}\left(\frac{\mu_{1}}{\mu_{2}}\right)\approx \sqrt{\frac{1}{n}}\sqrt{\frac{{s_{1}}^{2}{\mu_{2}}^{2}}{{\mu_{1}}^{4}}+\frac{{s_{2}}^{2}}{{\mu_{1}}^{2}}}$$

where μ_1 _and μ_2 _are the average IPD values of the modified and control, s_1 _and s_2 _are their standard deviations and n is the lower sequencing coverage of the two samples.

## List of Abbreviations Used

5hC: 5-hydroxycytosine; 5hmU: 5-hydroxymethyluracil; 5hU: 5-hydroxyuracil; 1mA: 1-methyladenine; 6mA: N6-methyladenine; 8oxoA: 8-oxoadenine; 8oxoG: 8-oxoguanine; BPDE: benzo[a]pyrene diol epoxide; IPD: interpulse duration; O4mT: O4-methylthymine; O6mG: O6-methylguanine; PW: pulse width; SMRT: Single-Molecule, Real-Time;

## Competing interests

The authors are full-time employees at Pacific Biosciences, a company commercializing single-molecule, real-time nucleic acid sequencing technologies.

## Authors' contributions

TC conceived of the study, made the SMRTbell templates, and assisted in drafting of the manuscript. KS carried out SMRT sequencing and data acquisition. ST participated in the design of the study and manuscript drafting. JK participated in the design and coordination of the study, made the figures, and drafted the manuscript. All authors read and approved the final manuscript.

## Supplementary Material

Additional File 1 **DNA template constructs used in this study**. The control template (top) contains annotations for the different oligonucleotides (Additional File 4) that make up the SMRTbell DNA template. Click here for file

Additional File 2 **Read termination induced by thymine glycol**. The first instance of thymine glycol in the DNA template at which sequencing reads end is highlighted with a black bar and underlined sequence label. Click here for file

Additional File 3 **Animation of the DNA polymerase catalytic cycle during SMRT sequencing**. The movie shows the binding of a phospholinked nucleotide, incorporation, and release of the pyrophosphate-linker-fluorophore reaction product. A hypothetical damaged DNA base is highlighted in red, and is moved into the active site following the phospholinked nucleotide incorporation and polymerase translocation. The animation highlights the close contact of the polymerase over an extended region with the nucleic acid: the incoming DNA template (positions -3 to zero) and nascent double-stranded DNA (positions +1 to approximately +7). The polymerization dynamics can be altered by the presence of DNA base modifications throughout this region. The animation is based on an in-house crystal structure and pdb structure 2PZS, followed by a brief energy minimization in PyMOL. The dye and linker are modeled solely for indicating their structures and do not reflect their real conformations and positions. Click here for file

Additional File 4 **Oligonucleotides and primers used to generate SMRTbell DNA templates**. All sequences are listed 5' to 3'. All oligonucleotides contain 5' phosphate groups. The primer binding site in the "Hairpin-Right" sequence is underlined. Click here for file
