# Supplementary material for: Direct Detection and Sequencing of Damaged DNA Bases
Source: Genome Integr. 2011 Dec 20;2:10. doi: 10.1186/2041-9414-2-10 (PMC3264494; doi:10.1186/2041-9414-2-10)
Supplement: Additional File 1 — DNA template constructs used in this study. The control template (top) contains annotations for the different oligonucleotides (Additional File 4) that make up the SMRTbell DNA template. [file 2041-9414-2-10-S1.PDF]

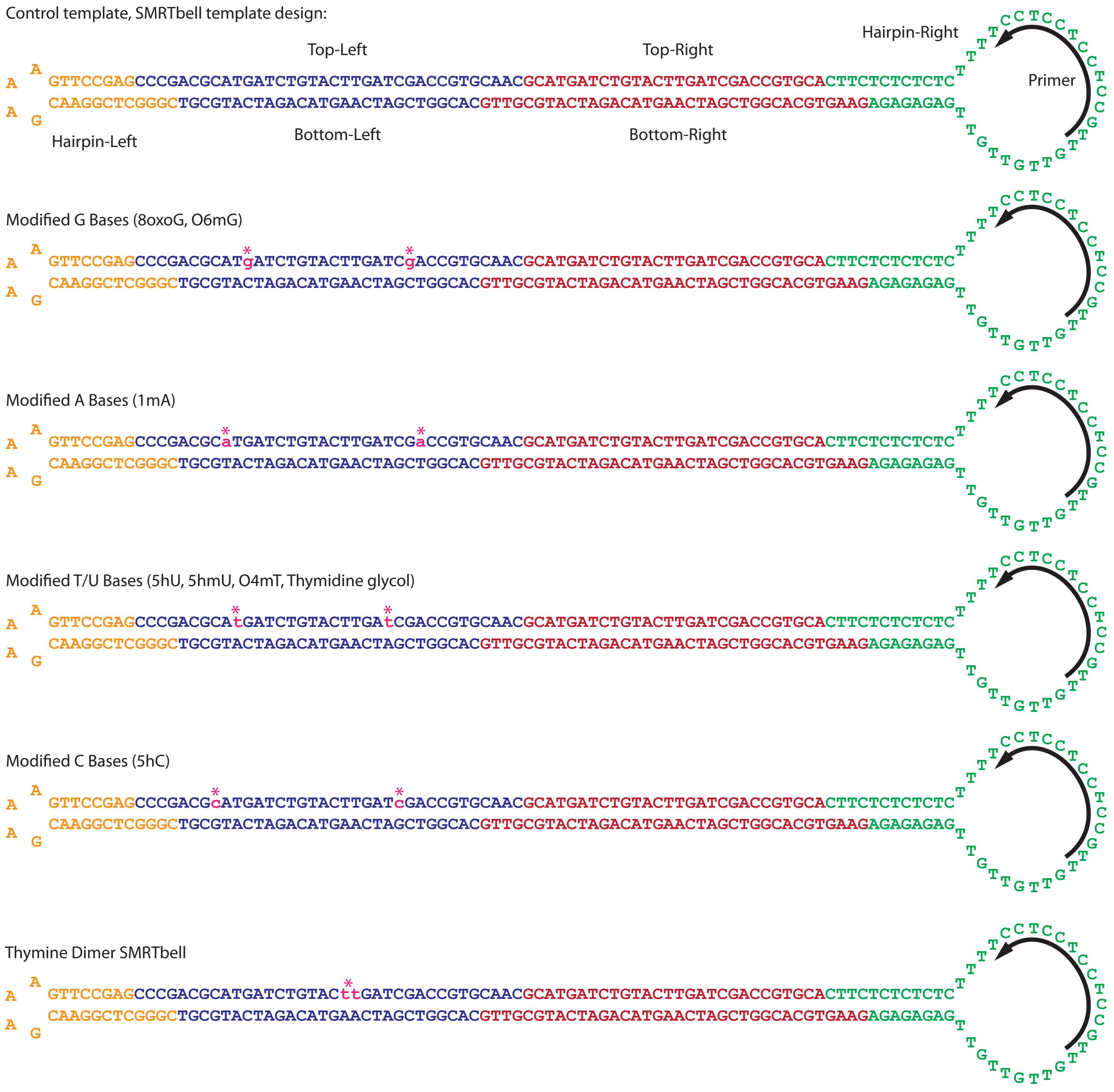

Additional File 1
