# Supplementary material for: Direct Detection and Sequencing of Damaged DNA Bases
Source: Genome Integr. 2011 Dec 20;2:10. doi: 10.1186/2041-9414-2-10 (PMC3264494; doi:10.1186/2041-9414-2-10)
Supplement: Additional File 2 — Read termination induced by thymine glycol. The first instance of thymine glycol in the DNA template at which sequencing reads end is highlighted with a black bar and underlined sequence label. [file 2041-9414-2-10-S2.PDF]

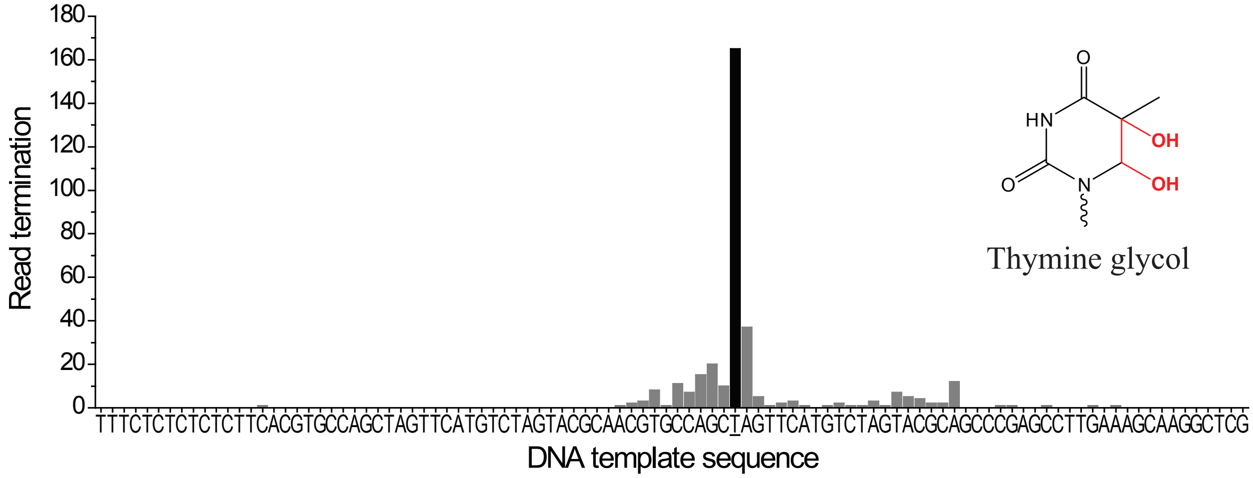

Additional File 2
